# Human gut‐associated lymphoid tissue: A dynamic hub propagating modulators of inflammation

**DOI:** 10.1002/ctm2.1417

**Published:** 2023-09-21

**Authors:** Sahil Jain, Mats Bemark, Jo Spencer

**Affiliations:** ^1^ Louise Coote Lupus Unit Guy's and St Thomas' NHS Foundation Trust London UK; ^2^ Department of Microbiology and Immunology University of Gothenburg Gothenburg Sweden; ^3^ School of Immunology and Microbial Sciences King's College London London UK

**Keywords:** B cells, human gut, inflammation, lymphoid tissue

The populations of bacteria that comprise the gut microbiota are associated with disease in many ways, but none of them are understood with any clarity at the level of specific taxa.^1^ We know, for example, that microbial diversity is good for health, and that bacterial species that can generate the short‐chain fatty acids that promote immune suppression are in general good for us (as described below), but there are no consistently identified bacterial species involved. Still, microbes in the gut lumen chronically stimulate gut‐associated lymphoid tissues (GALT). These relatively small though abundant foci of cells can be major regulators of intestinal and systemic immune homeostasis. Thus, an important role of the microbiota and their associated antigens is to maintain GALT in an activated, yet balanced, state.^2^ Here, we discuss some ways in which the chronically activated GALT, that are comprised mostly of B cells, can impact intestinal and systemic inflammatory diseases.

## 1 WHAT IS GALT?

**FIGURE 1:**
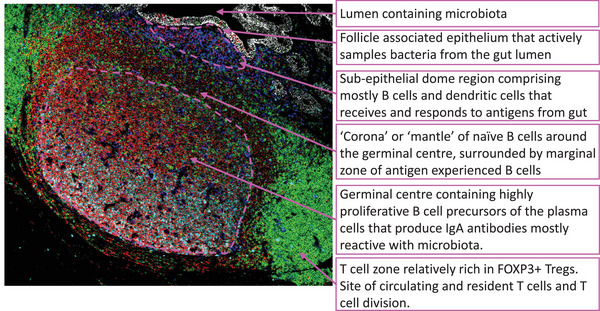
Image from a transverse section through a block of formalin fixed, paraffin embedded human appendix identifying an example of GALT. Section is stained to identify E‐Cadherin (epithelial monolayer: white), CD20 (B cells: red), CD3 (T cells: green), CD11c (dendritic cells: dark blue) and Ki67 (dividing cells: cyan blue). The different cellular contents of and functions of the zones are indicated in boxes. The approximate boundaries of the subepithelial dome and the germinal centre are indicated with dashed lines in magenta.

Examples of human GALT are the organized lymphoid tissues in the vermiform appendix, the Peyer's patches in the terminal ileum, colonic isolated lymphoid follicles and rectal follicles that all have similar structural features.^2^ They are all intrinsically linked in structure and function to the follicle‐associated epithelium that actively samples the contents of the gut lumen and transfers it to the region of lymphoid tissue just below the epithelial layer which is referred to as the sub‐epithelial dome (Figure 1). As a result, GALT are stimulated by microbiota throughout life. Activation of lymphocytes in GALT results in the generation of precursors of effector cells, including cytokine‐producing T cells and IgA plasma cells, that will migrate from the tissue in lymphoid vessels eventually reaching the bloodstream. IgA plasma cells generated in this way will have binding specificity for gut bacteria.^3^ Activated precursors to the effector cells become ‘imprinted’ with the ability to home to the gut by, for example, induction of integrin α4β7 and then enter the blood via the lymphatic system.^4^ α4β7 binds to the glycoprotein antigen MAdCAM1 that protrudes into the lumen of the small blood vessels that vascularize the gut. MAdCAM ‘hooks’ cells in blood expressing integrin α4β7 and recruits them into sites of immune cell effector function. For the B cell arm of the immune response, these are the precursors of the IgA plasma cells that secrete dimeric IgA. The drug vedolizumab that blocks the binding of α4β7 to MAdCAM1 has shown efficacy for the treatment of ulcerative colitis supporting the validity of this model and the importance of cell homing to the gut in disease progression.^5^

## 2 PLASMA CELLS DERIVED FROM GALT IN HEALTH AND INFLAMMATION

IgA is adapted to work efficiently in the gut lumen. Through interactions with the J chain that links IgA monomers into dimers, it is transported with the secretory component across enterocytes into the gut lumen. As a dimeric antibody structure, it has four binding arms and can, therefore, cross‐link bacteria, potentially forming a lattice that facilitates processes, such as immune exclusion.^2^ It does not fix complement, a factor that contributes to inflammatory responses and, therefore, IgA is not proinflammatory in the gut milieu. Also, as IgA primarily exerts its functions in the acellular environment of the gut lumen, it does not interact with phagocytes through Fc’ receptors.

A study that used deep multiplexed methods to investigate the immune drivers associated with ulcerative colitis severity observed that a major parameter associated with the disease is IgG.^6^ Biases towards IgG‐producing plasma cells in inflammatory bowel disease (IBD), which also include IgG subclass biases, have been acknowledged for decades.^2^ Cutting‐edge methods to study cells and tissues now give us the ability to identify the important detail among the many facts we already know. Thus, the effectiveness of vedolizumab^5^ could be related to the blocking of homing of IgG‐secreting effector cells as well as cytokine‐secreting effectors.

## 3 GALT AND THE DEVELOPMENT OF IMMUNE REGULATORS

Although the involvement of GALT in the biology of regulatory B and T cells is unclear, Treg cells can be observed at a relatively high frequency in the T cell zones of GALT. Two types of Treg cells have been described: thymus‐derived natural and peripherally induced. Both are present in GALT in mice, with T follicular regulatory cells being present among thymus‐derived Treg cells with a memory phenotype. Treg cells are crucial to maintaining intestinal homeostasis in humans and mouse models.^7^ GALT are likely key microenvironments for Treg activation and imprinting. In a mouse model, an antibody to β7 integrin exacerbated colitis by inhibiting the homing of Treg cells to the gut lamina propria.^8^

Regulatory B (Breg) cells are a population of B cells that produce IL‐10 and other immunoregulatory cytokines. Although several cell surface profiles have been linked to Breg cells, such as B cell immaturity with high expression of CD38 and CD24, as well as plasma cell differentiation, these are neither consistent nor universally accepted. However, cytokine‐producing B cells are undoubtedly important: reduction in Breg cell function has been described in mouse models of arthritis and in patients with rheumatoid arthritis.^9^ In mouse models, Breg cells producing immunosuppressive IL‐10 mature in the spleen dependent on signals derived from the gut microbiota. The mechanism driving this protection is the production of the short‐chain fatty acid butyrate by bacterial fermentation of dietary fibre. Butyrate then regulates the availability of the serotonin‐derived metabolite 5‐hydroxyindole‐3‐acetic acid that can bind the aryl hydrocarbon receptor on the surface of Breg cells to promote their anti‐inflammatory properties. Butyrate also promotes regulatory T (Treg) cell induction in mice. It is known to affect macrophage function and the properties of the bacterial populations themselves in human cells in vitro and mice in vivo. Thus, Breg cells belong to a network of events driven by the microbiota in a butyrate‐dependent way that modulates local and systemic immune responses.

Supporting the importance of butyrate derived from microbiota and its interactions in human disease, stool from patients with rheumatoid arthritis and mice with joint inflammation had reduced levels of butyrate compared with stool from healthy controls.^9^ It was suggested that modulation of this pathway could be a potential therapeutic intervention to limit joint inflammation.

## 4 DNASE1L3 IN GALT—A LINK BETWEEN INTESTINAL IMMUNITY AND AUTOIMMUNITY

Recently published work identified that within the GALT, the secreted DNAse DNASE1L3 is most abundantly produced in the subepithelial dome, where it presumably digests DNA derived from sampled gut microbiota.^10^ This area did not contain abundant apoptotic cells, and thus human DNASE1L3 is not associated with the digestion of cellular debris to the same extent as it is in mice. Defects in DNASE1L3 function, either by loss of function mutations or through functionally inhibitory autoantibodies, result in the generation of anti‐DNA antibodies. Thus, if DNASE1L3 is inactive, it is possible that the production of anti‐DNA antibodies could be antigenically driven by bacterial DNA in GALT. This has deep relevance to the pathogenesis of systemic lupus erythematosus and lupus nephritis; resolving the relevance of this novel finding will be an important challenge.

## 5 CONCLUSION

Thus, while bacteria that comprise the gut microbiota per se are important, beyond the potential of some species to produce butyrate, individual species may not hold the key to understand links with disease pathogenesis. Rather, we propose that a major function of microbiota and its importance in disease is through its ability to modulate GALT function.
